# High Diabetes Prevalence among Tuberculosis Cases in Kerala, India

**DOI:** 10.1371/journal.pone.0046502

**Published:** 2012-10-15

**Authors:** Shibu Balakrishnan, Shibu Vijayan, Sanjeev Nair, Jayasankar Subramoniapillai, Sunilkumar Mrithyunjayan, Nevin Wilson, Srinath Satyanarayana, Puneet K. Dewan, Ajay M. V. Kumar, Durai Karthickeyan, Matthew Willis, Anthony D. Harries, Sreenivas Achuthan Nair

**Affiliations:** 1 Department of Tuberculosis, Office of the WHO Representative to India, New Delhi, India; 2 International Union Against TB and Lung Diseases, South East Asia Regional Office, New Delhi, India; 3 Department of Respiratory Medicine, Government Medical College, Thiruvananthapuram, Kerala, India; 4 State TB Cell, Directorate of Health Services, Thiruvananthapuram, Kerala, India; 5 Epidemic Intelligence Service Centers for Disease Control and Prevention, Division of TB Elimination, Atlanta, Georgia, United States of America; 6 International Union Against Tuberculosis and Lung Disease, Paris, France; 7 London School of Hygiene and Tropical Medicine, London, United Kingdom; University College Dublin, Ireland

## Abstract

**Background:**

While diabetes mellitus (DM) is a known risk factor for tuberculosis, the prevalence among TB patients in India is unknown. Routine screening of TB patients for DM may be an opportunity for its early diagnosis and improved management and might improve TB treatment outcomes. We conducted a cross-sectional survey of TB patients registered from June–July 2011 in the state of Kerala, India, to determine the prevalence of DM.

**Methodology/Principal Findings:**

A state-wide representative sample of TB patients in Kerala was interviewed and screened for DM using glycosylated hemoglobin (HbA1c); patients self-reporting a history of DM or those with HbA1c ≥6.5% were defined as diabetic. Among 552 TB patients screened, 243(44%) had DM – 128(23%) had previously known DM and 115(21%) were newly diagnosed - with higher prevalence among males and those aged >50years. The number needed to screen(NNS) to find one newly diagnosed case of DM was just four. Of 128 TB patients with previously known DM, 107(84%) had HbA1c ≥7% indicating poor glycemic control.

**Conclusions/Significance:**

Nearly half of TB patients in Kerala have DM, and approximately half of these patients were newly-diagnosed during this survey. Routine screening of TB patients for DM using HbA1c yielded a large number of DM cases and offered earlier management opportunities which may improve TB and DM outcomes. However, the most cost-effective ways of DM screening need to be established by futher operational research.

## Introduction

Diabetes Mellitus (DM) almost triples the risk of developing tuberculosis (TB) [1]–[4]. India, the nation with the highest number of TB cases in the world, is also undergoing epidemic growth in DM rates. The estimated prevalence of DM in India in 2010 was 51 million and this is projected to increase to 70 million by 2025 [5], [6]. In India, 15% of pulmonary tuberculosis cases have been estimated to be attributable to DM [7]. An analysis of nutrition and DM changes in India also suggests that increased DM prevalence between 1998 and 2008 contributed to an increase in the total number of TB cases in the country which exceeded the rate of population growth in the same time period [8]. These findings highlight the impact of the DM epidemic on TB incidence rates in the country.

A recent systematic review of the effect of DM on TB treatment outcomes showed that DM may delay sputum culture conversion, may increase the case fatality rate during treatment, and may increase relapse rates of TB after successful completion of treatment [9]. A study from Kerala shows that non-drug resistant TB patients with DM were more likely to fail first line TB treatment as compared to those without DM [10]. Therefore, active screening for DM among TB patients may provide an opportunity to identify previously undiagnosed diabetes and improve TB treatment outcomes through optimum diabetic care. However, neither international guidelines nor India's Revised National TB Control Programme (RNTCP) currently recommend active screening of TB patients for detection of DM.

DM is common in the south Indian state of Kerala (population 34.6 million), with an estimated community prevalence of 16% to 20% [11], [12]. We therefore conducted this study to determine the overall prevalence of DM among TB patients and to assess whether routine screening of TB patients for DM within a programme setting might yield previously undiagnosed DM cases, offering an opportunity for earlier detection and treatment of DM. The specific objectives of this study were to determine (i) the overall prevalence of DM (self-reported and newly diagnosed) among TB patients registered for treatment under RNTCP in Kerala state (ii) additional yield of previously unknown DM as measured by glycosylated haemoglobin (HbA1c) and the Number needed to screen (NNS) to find a new case of DM and (iii) the factors associated with DM among TB patients.

## Methods

### Study Design

This was a cross-sectional study of a sample of TB patients diagnosed and registered in 2011 in which self-report and HbA1c was used to diagnose DM.

### Study Setting

The study was conducted in Kerala, the southernmost state of India. In 2010, approximately 25,000 patients were diagnosed and treated through 73 TB reporting units statewide [13]. According to national guidelines [14], sputum smear microscopy is performed for all TB suspects. Patients are diagnosed with pulmonary TB (PTB) if at least one initial sputum sample contains acid fast bacilli. Smear negative PTB cases are diagnosed by chest radiograph subsequent to negative results for repeated sputum smear microscopy, after two weeks of the first results. Extra-pulmonary TB (EPTB) cases are diagnosed by a combination of histopathology, mycobacteriology and/or clinical features. All TB patients are treated with a rifampicin containing, fully intermittent (thrice weekly), standardized treatment regimen delivered under direct observation. Patients are registered for treatment in one of the designated TB units of the state and monitored for their treatment outcome. TB cases are further classified as per standard WHO definitions [15]. Kerala state has been proactive and ahead of the national efforts in providing diagnostic and treatment services for DM at secondary care level within its general health system at subsidized rates for the patients [16].

The national programme dealing with all non-communicable diseases including diabetes (NPCDCS for National programme for prevention and control of Cancer, Diabetes, Cardiovascular diseases and Stroke) is still in its early stages in India and is being piloted in 30 districts across the country with plans to extend it to 100 districts by the end of 2012. However, the Government of India is committed to achieve nationwide expansion in the 12th five year plan period (2012–17) [17]. In these pilot districts, diagnostic and treatment services for DM have been decentralized to primary care level, free of cost to the patients.

### Sample size and Sampling

**Figure 1:**
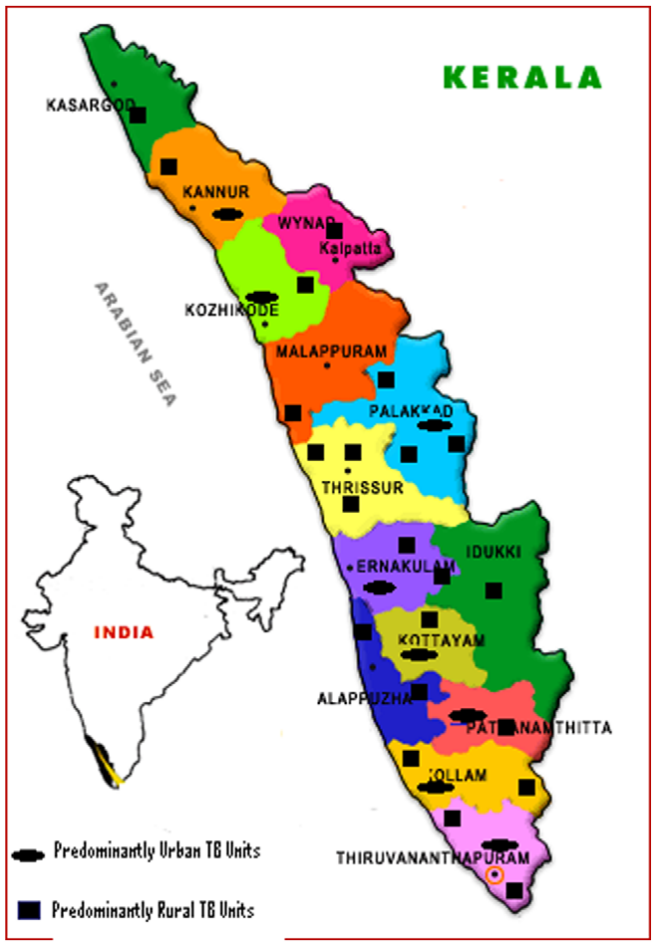
Sampled TB units in Kerala to estimate the prevalence of Diabetes Mellitus among TB patients.

All patients aged >15 years diagnosed and registered with any type of TB in Kerala's 73 TB Units between June and July 2011 were eligible for this study. The sample size for the study was calculated based on an estimated prevalence of DM in TB patients of 25% with a precision of 5% and a design effect of two. Cluster sampling method was chosen with each TB unit considered a cluster. Of the 73 TB Units, 30 were selected randomly to create a representative sample of the state. Of the TB units selected, 22 TB units were predominantly rural and 8 TB units were predominantly urban, in line with the expected rural-urban distribution of the population in the state of Kerala. The number of patients enrolled in each cluster was proportionate to number of TB cases notified from each selected TU. In each TB unit, consecutively registered TB patients were enrolled until the required sample was achieved. The names of the TB units, sample size planned from each cluster and sample size achieved are shown in the table. (Table 1. Details of the 30 TB Units (clusters) selected by cluster sampling for the study, Kerala, India, June–July 2011). The location of TB units selected is shown in the map. (Figure 1: Sampled TB units in Kerala to estimate the prevalence of Diabetes Mellitus among TB patients)

**Table 1:** Details of the 30 TB Units (clusters) selected by cluster sampling for the study, Kerala, India, June–July 2011.

Name of the TB unit	Type of TB Unit	Population	Adult TB Case Notification October to December 2009	Sample size proposed	Sample Size achieved
Adoor	Rural	525924	100	32	30
Alathur	Rural	431834	75	21	21
Chavakkad	Rural	561856	69	19	12
Cherthala	Rural	554693	74	25	25
Chirayinkeezhu	Rural	536603	71	18	0
Chittur	Rural	448880	107	30	30
Kochi	Urban	498868	84	25	25
Irinjalakkuda	Rural	548386	65	22	20
Kasaragod	Rural	502522	62	19	8
Karunagappally	Rural	467111	82	27	27
Kollam_DTC	Urban	803899	120	32	25
Kothamangalam	Rural	248208	39	11	11
Kottayam	Urban	681484	124	33	34
Koyilandy	Urban	520464	70	22	22
Mavelikkara	Rural	561102	100	29	29
Mukkam	Rural	447704	47	16	16
Neyyattinkara	Rural	508572	64	19	0
Ottapalam	Rural	485813	92	24	24
Painavu	Rural	259416	18	7	7
Pala	Rural	424809	51	17	17
Palakkad_DTC	Urban	545474	100	36	36
Payyannur	Rural	463532	53	12	12
Peroorkada	Urban	500110	60	17	0
Perumbavoor	Rural	420324	70	21	13
Ponnani	Rural	423191	44	14	15
Punalur	Rural	483612	78	25	25
Thalassery	Urban	504638	64	20	16
Thiruvalla	Urban	468174	60	17	17
Wadakkanchery	Rural	521066	81	24	19
Wayanad_DTC	Rural	265509	35	15	16
Total				650	552

### Data Collection and analysis

Data were collected using a structured questionnaire to elicit demographic and clinical variables including a history of diabetes. For patients consenting to DM screening, blood samples were collected within one month of diagnosis of TB and glycosylated hemoglobin (HbA1c) levels were measured from whole blood using High Pressure Liquid Chromatography on a Biorad D10 Machine according to standard procedures, an NGSP (National Glycohemoglobin Standardization Program) accepted method with low co-efficient of variation and less likely to be affected by interfering substances. An HbA1c value of 6.5% or above was considered diagnostic of DM, in line with recommendations of American Diabetes Association and WHO [18]–[20]. For patients with self-reported DM, a glycosylated hemoglobin (HbA1c) level >7% was taken to indicate poorly controlled disease [21]. We chose to measure glycosylated hemoglobin level as a tool to diagnose DM as it has multiple advantages over traditional fasting plasma glucose measurement. First, it is a valid method and is now recommended by all the leading technical agencies around the globe. Second, it is very convenient for the patient as blood sample can be drawn at the time of initial visit and does not require patient preparation in terms of being in the fasting state for 8 hours, which often places unnecessary burden on very sick patients. Third, it is least influenced by temporary changes in the levels of blood glucose due to various factors including acute infection like Tuberculosis.

We used Epi Info 7 (Version 7.0.8.0) to analyze the data and performed a complex sample analysis to account for the cluster sampling methodology [22]. The main outcomes for analysis were the number and proportion (with 95% confidence interval) of TB patients with a diagnosis of DM (previously known and newly diagnosed), stratified by age, sex, place of residence and type of TB. Odds ratios with 95% confidence intervals have been used to describe the differences between groups. Multivariate analysis using logistic regression was done to calculate adjusted odds ratios. Only variables that were statistically significant (p<0.05) in bivariate analysis were considered for multivariate analysis.

### Ethics approval

Written informed consent was taken from all the study participants and from the parents in case of minors, ie, for participants of age 15 to 18 years, before enrollment in the study and those TB patients who were diagnosed to be having DM were linked to standard care available in the government health system which included counseling, lifestyle modification, specialist's consultation, anti-diabetic medications including insulin provided free of cost and management of complications. The study was approved by the ethics committee of National Tuberculosis Institute (NTI), Bangalore, India and the Ethical Advisory Group of The International Union against Tuberculosis and Lung Diseases (The Union).

## Results

The overall prevalence of DM among TB patients disaggregated by age, sex, residence and type of TB is shown in Table 2 (Table 2 Prevalence of Diabetes Mellitus among tuberculosis patients registered for treatment in the state of Kerala, India, June–July 2011). There were 552 patients [mean (SD) age 46 (15) years] enrolled in the study. Three quarters of the patients were male and the majority were from a rural setting. New TB patients constituted 86% of all patients, and of these 65% had smear-positive pulmonary TB (Table 2).

**Table 2:** Prevalence of Diabetes Mellitus among tuberculosis patients registered for treatment in the state of Kerala, India, June–July 2011.

Characteristic	Number of TB patients whose DM status was ascertained	Number with DM	Prevalence of DM (95% CI)
**Total**	552	243	44.0 (38.8–49.3)
**Sex**			
Male	420	208	49.5 (43.6–55.4)
Female	132	35	26.5 (19.1–33.9)
**Age (years)**			
15–24	57	4	7.0 (0.6–13.4)
25–34	70	15	21.4 (10.6–32.2)
35–44	114	44	38.6 (29.1–48.1)
45–54	134	74	55.2 (46.5–63.9)
55–64	106	67	63.2 (51.7–74.7)
≥65	71	39	54.9 (42.1–67.7)
**Residence**			
Urban	107	51	47.6 (35.8–59.5)
Rural	445	192	43.1 (37.9–48.4)
**Type of TB**			
New Smear Positive Pulmonary TB	307	157	51.1 (44.3–57.9)
New Smear Negative Pulmonary TB	37	11	29.7 (17.7–41.7)
New Extra-pulmonary TB	128	36	28.1 (18.4–37.8)
Relapse	35	20	57.1 (40.8–73.5)
Treatment after Failure	19	9	47.3 (24.5–70.2)
Treatment after Default	26	10	38.4 (18.3–58.5)

TB-Tuberculosis; DM- Diabetes Mellitus; CI-Confidence Interval; Number with DM includes self-reported, previously known cases and those newly diagnosed with a glycosylated hemogolobin level of ≥6.5%.

Of 552 TB patients assessed, 243 (44%) were found to have DM (Table 2). The prevalence of DM was higher among males, those aged 50 years and above and among smear positive TB cases, particularly among Relapse TB cases.

Of 243 diabetics, 128 (23%) TB patients self-reported a previous diagnosis of DM and 115 (21%) were newly diagnosed to have DM, an additional yield of about 47%. (Table 3 Additional Yield of new cases of Diabetes Mellitus and Number Needed to Screen to diagnose a new case of DM among tuberculosis patients in the state of Kerala, India, June–July 2011). After excluding previously known DM, the number of TB patients that needed to be screened (NNS) to find one new case of DM was approximately 4 (Table 3). The NNS ranged between 2–6 across all categories except for those aged 15–24 years in whom it was 53.

**Table 3:** Additional Yield of new cases of Diabetes Mellitus and Number Needed to Screen to diagnose a new case of DM among tuberculosis patients in the state of Kerala, India, June–July 2011.

Characteristic	Number of TB patients whose DM status was ascertained [a]	Number with previously known DM [b]	Number of DM newly diagnosed [c]	Additional Yield [c/(b+c)*100]	Number needed to screen (NNS) [(a−b)/c]
**Total**	552	128	115	47%	3.7
**Sex**					
Male	420	108	100	48%	3.1
Female	132	20	15	43%	7.5
**Age (years)**					
15–24	57	3	1	25%	52.6
25–34	70	4	11	73%	6.0
35–44	114	19	25	57%	3.8
45–54	134	44	30	41%	3.0
55–64	106	38	29	43%	2.3
≥65	71	20	19	49%	2.6
**Residence**					
Urban	107	37	14	27%	5.0
Rural	445	91	101	53%	3.5
**Type of TB**					
New Smear Positive Pulmonary TB	307	87	70	45%	3.1
New Smear Negative Pulmonary TB	37	4	7	64%	4.7
New Extra-pulmonary TB	128	15	21	58%	5.3
Relapse	35	12	8	40%	3.3
Treatment after Failure	19	7	2	22%	6.0
Treatment after Default	26	3	7	70%	3.3

TB-Tuberculosis; DM- Diabetes Mellitus; NNS-Number needed to screen is defined as the reciprocal of the prevalence of newly diagnosed DM after excluding self-reported previously known cases of DM.

Results of the bivariate and multivariate analysis are presented in Table 4. (Table 4 Factors associated with Diabetes Mellitus among tuberculosis patients registered for treatment in the state of Kerala, India, June–July 2011). Male sex and age above 50 years were significantly associated with a diagnosis of DM and remained independently associated even after adjusting for other variables in a logistic regression model (Table 4).

**Table 4:** Factors associated with Diabetes Mellitus among tuberculosis patients registered for treatment in the state of Kerala, India, June–July 2011.

Characteristic	Unadjusted Odds Ratio (95% CI)	Adjusted Odds Ratio (95% CI) #
**Sex**		
Male	**2.7 (1.8–4.0)** *	**2.3 (1.5–3.6)** *
Female	Ref	Ref
**Age (years)**		
≥50	**3.0 (2.2–4.3)** *	**2.8 (2.0–4.0)** *
<50	Ref	Ref
**Residence**		
Urban	1.2 (0.7–1.9)	NA
Rural	Ref	
**Type of TB**		
New	Ref	NA
Previously treated	1.2 (0.8–2.0)	

TB-Tuberculosis; DM- Diabetes Mellitus; NA-Not applicable.

* *P* value less than 0.05;

# - adjusted for age and sex using Logistic regression.

Of 128 patients with a self-reported history of DM, 107 (84%) (95% CI 76.0–89.5) had a glycosylated haemoglobin level equal to or more than 7%, indicating poorly controlled DM.

## Discussion

We found a high prevalence of DM amongst TB patients registered under RNTCP in the state of Kerala, India and this was significantly associated with male sex and age above 50 years. About one quarter of the patients gave a self-reported history of DM, and this was more common in those living in an urban environment and aged 50 years and above. This association with urban residence may indicate lifestyle practices that predispose to DM or better access to diagnosis of DM in urban settings. Of concern was the finding that the majority of these self-reported patients had poorly controlled DM, defined as glycosylated haemoglobin levels greater than or equal to 7%. This is as per the recommended glycemic goals for nonpregnant adults by American Diabetes Association which is HbA1c level of <7% corresponding to a preprandial capillary plasma glucose 70–130 mg/dl (3.9–7.2 mmols/l) to define the control status of diabetes [21]. In those with no self-reported history of DM, over one quarter had glycosylated haemoglobin levels greater than or equal to 6.5%, which is recognised by WHO as diagnostic of DM. In total, nearly half (44%, 95% CI 38.8–49.3) of the TB patients in Kerala were found to have DM, the highest reported prevalence of DM among TB patients to date. Furthermore 115 (21%) TB patients were found to have previously-undiagnosed DM. These findings are an alarming signal that DM should be considered and investigated in all TB patients in Kerala. This is highly justified given the high returns in terms of additional yield of newly diagnosed DM cases found in our study. The number needed to screen (NNS) to find one new case of DM was just four overall and ranged mainly from two to six, with the exception of the age group 15–24 years in whom it was about 50. This indicates that the strategy of routine screening of all TB patients for DM is likely to be cost-effective.

The high prevalence of DM amongst TB patient in the state follows a similar pattern of high prevalence of DM in the general population. Prevalence of DM as reported by the community serveys using blood sugar estimation among the general adult population in the state is high (16–20%) and is higher than that of the rest of the country [12]. On the other hand, prevalence of tuberculosis transmission, is less in Kerala compared to the rest of the country [23]. TB patients of Kerala are older than those in the rest of India and DM could be one of the important factors influencing tuberculosis incidence in Kerala.

The strengths of this study are that large numbers of TB patients were consecutively enrolled in 30 reporting units across Kerala state, India, and therefore this cohort is probably representative of the state. However, this may not be representative of the situation in India because the prevalence of DM in Kerala is higher than that reported nationally and from other states in the country [24]. Another strength is that glycosylated haemoglobin levels were measured on all patients. This measurement provides an index of blood glucose levels over a period 2–3 months and is not subject to the rapid swings that can occur with random and fasting blood glucose measurements. Blood glucose levels at a specific moment in time can reflect recent diet and may also be affected by physiologic stress such as active tuberculosis as well as anti-TB treatment. Possible limitations of the study include the lack of information on factors such as alcohol intake or use of other drugs which might affect glycosylated haemoglobin levels. As an infectious disease, TB may temporarily elevate blood glucose levels resulting in false-positive diagnoses of diabetes if investigations are done too early. Several observational studies have shown that prevalence of hyperglycemia decreases with TB treatment and hence more research with multiple measurements during the course of treatment is required to define the optimal timing of screening for DM [25].

We know of no other prospective study that has measured glycosylated haemoglobin levels in patients with TB in India. There have been studies conducted in India assessing the prevalence of DM in TB patients by meausrement of random and fasting blood glucose levels, and these have found prevalence levels that vary from 2% to 20% [26]–[30]. Routine screening of TB patients for DM using random and fasting blood glucose level measurements by TB service providers in Kerala had reported a prevalence of 26% among TB patients of age 15 years or more [31]. Routine screening of multi drug resistant TB patients treated under RNTCP in Kerala with fasting and post meal blood glucose levels showed a prevalence of DM of 40%. A study similar to our study measuring glycosylated haemoglobin by Blanca et. al in Texas where community prevalence of DM was 19% reported a 39% prevalence of DM among TB patients [32].

### Policy implications

This study has important policy implications. First, it is advisable to perform similar studies in different states in India using the same methodology in order to get a good measure of DM prevalence in TB patients across the country. Second, as is the case with HIV in sub-Saharan Africa, it will be essential to ask TB patients routinely about whether or not they have DM. If patients do give a self-reported history of DM, then they must be referred to appropriate DM care as most of the patients in this study had poorly controlled diabetes disease. TB patients with DM have an increased risk of death and this may be reduced through better DM control and treatment. Third, the study suggests it is important to routinely and actively screen all TB patients for DM, including those who do not self-report the disease. The major question is how to do this most effectively and efficiently [33]. Although glycosylated haemoglobin would appear to be the best way to screen as it is relatively unaffected by active TB and anti-TB drugs, it is a costly laboratory investigation which might be difficult to scale up and to use in peripheral health facilities. This requires a cost-effectiveness analysis. Fourth, there is limited information on various aspects of interaction between TB and DM including optimal tools and strategies for collaboration. This urgently calls for research - both clinical and operational research - to understand these interactions better and optimize the intervention strategies [25]. Finally, it is important to have a well functioning non-communicable disease control programme linked with the TB control programme in settings where the prevalence of DM among TB patients is high. The recently published WHO-Union collaborative framework for care and control of DM-associated tuberculosis is a good template for action and should be considered for implementation by the national programmes [25].

## Conclusions

In conclusion, this study has shown a high prevalence of DM in patients with active TB, both for self-reported DM and for patients in whom the disease was not suspected, and supports routine screening of TB patients for DM. However, the best way of doing this needs to be established by futher operational research studies.
